# The Wild Mouse (*Micromys minutus*): Reservoir of a Novel *Campylobacter jejuni* Strain

**DOI:** 10.3389/fmicb.2019.03066

**Published:** 2020-01-14

**Authors:** Junhyung Kim, Jae-Ho Guk, Seung-Hyun Mun, Jae-Uk An, Woohyun Kim, Soomin Lee, Hyokeun Song, Je Kyung Seong, Jun Gyo Suh, Seongbeom Cho

**Affiliations:** ^1^Research Institute for Veterinary Science, College of Veterinary Medicine, Seoul National University, Seoul, South Korea; ^2^Department of Medical Genetics, College of Medicine, Hallym University, Chuncheon, South Korea

**Keywords:** *Campylobacter jejuni*, wild mouse, transmission cycle, whole genome sequencing, comparative genomic analysis

## Abstract

*Campylobacter jejuni* is one of the most common zoonotic pathogens worldwide. Although the main sources of human *C. jejuni* infection are livestock, wildlife can also affect *C. jejuni* transmission in humans. However, it remains unclear whether wild mice harbor *C. jejuni* and are involved in the “environment–wildlife–livestock–human” transmission cycle of *C. jejuni* in humans. Here, we characterized *C. jejuni* from wild mice and identified genetic traces of wild mouse-derived *C. jejuni* in other hosts using a traditional approach, along with comparative genomics. We captured 115 wild mice (49 *Mus musculus* and 66 *Micromys minutus*) without any clinical symptoms from 22 sesame fields in Korea over 2 years. Among them, *M. minutus* were typically caught in remote areas of human houses and *C. jejuni* was solely isolated from *M. minutus* (42/66, 63.6%). We identified a single clone (MLST ST-8388) in all 42 *C. jejuni* isolates, which had not been previously reported, and all isolates had the same virulence/survival-factor profile, except for the plasmid-mediated *vir*B11 gene. No isolates exhibited antibiotic resistance, either in phenotypic and genetic terms. Comparative-genomic analysis and MST revealed that *C. jejuni* derived from *M. minutus* (strain SCJK2) was not genetically related to those derived from other sources (registered in the NCBI genome database and PubMLST database). Therefore, we hypothesize that *C. jejuni* from *M. minutus* is a normal component of the gut flora following adaptation to the gastro-intestinal tract. Furthermore, *M. minutus*-derived *C. jejuni* had different ancestral lineages from those derived from other sources, and there was a low chance of *C. jejuni* transmission from *M. minutus* to humans/livestock because of their habitat. In conclusion, *M. minutus* may be a potential reservoir for a novel *C. jejuni*, which is genetically different from those of other sources, but may not be involved in the transmission of *C. jejuni* to other hosts, including humans and livestock. This study could form the basis for further studies focused on understanding the transmission cycle of *C. jejuni*, as well as other zoonotic pathogens originating from wild mice.

## Introduction

*Campylobacter jejuni* is one of the most common zoonotic pathogens worldwide, and human infections with this pathogen have increased in both developing and developed countries (Kaakoush et al., 2015). In particular, *C. jejuni* directly causes gastrointestinal diseases such as diarrhea and abdominal pain, and it can also lead to late complications such as neurological diseases, including Guillain–Barre and Miller–Fisher syndromes in humans (Nachamkin et al., 1998). The main sources of *C. jejuni* transmission in humans are livestock, including poultry and cattle, and humans in particular are easily exposed to *C. jejuni* during the handling or ingestion of poultry (Bronowski et al., 2014). Environmental sources are also reservoirs for *C. jejuni* infections in humans, and transmission through contaminated water and soil is prevalent (Gras et al., 2012). In addition, wildlife species can serve as potential reservoirs for *C. jejuni* infection in humans (Waldenström et al., 2007). Therefore, humans, livestock, environmental sources, and wildlife form complex interactions that contribute to *C. jejuni* infection and constitute a transmission cycle. Studies of *C. jejuni* in various reservoirs are needed to better understand the potential for *C. jejuni* transmission and the impact that such transmission could have on human health.

Studies on *C. jejuni* in wildlife have been conducted mainly with wild birds, since they can contribute to *C. jejuni* infections in humans and livestock (French et al., 2009; Weis et al., 2016). In addition to research on wild birds, *C. jejuni* infections in wild mice are also important to study, since wild mice are prevalent, come in close contact with humans, and are known carriers of various pathogens (Kruse et al., 2004; Han et al., 2015). However, few studies have examined *C. jejuni* infection in wild mice, and no studies evaluating the effects of wild mice on human *C. jejuni* infection have been reported. Only one study provided an estimate of *C. jejuni-*transmission events between rodents and pigs on the same farm (Meerburg et al., 2006). Thus, the possibility of wild mice being a reservoir of *C. jejuni* and playing a role in the transmission of *C. jejuni* between wild mice and other hosts remains unclear, and no studies have shown that wild mice (similarly to wild birds) contribute to human *C. jejuni* infection (Meerburg and Kijlstra, 2007).

The objectives of this study were to determine whether wild mice harbor *C. jejuni* in their normal gut and whether they are involved in the “environment–wildlife–livestock–human” transmission cycle of *C. jejuni* in human infections. To characterize *C. jejuni* from wild mice, *C. jejuni* was isolated from wild mice (*Mus musculus* and *Micromys minutus*) in 22 sesame fields over the course of 2 years. The clonal distribution of *C. jejuni* isolates from these wild mice was identified, and the virulence/survival-factor profiles and antibiotic-resistance patterns of all isolates were determined. Furthermore, to investigate a genetic trace of *C. jejuni* transmission between wild mice and other hosts, the genetic relatedness of *C. jejuni* between wild mice and other sources was compared using comparative-genomic analysis.

## Materials and Methods

### Sampling of Wild Mice and Isolation of *C. jejuni*

**FIGURE 1:**
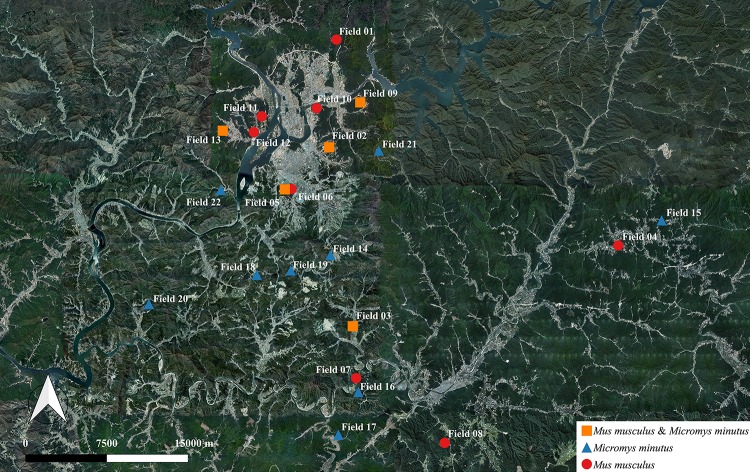
Information regarding the habitat of wild mice, *Mus musculus* and *Micromys minutus*. We captured 115 wild mice, including 49 *M. musculus* and 66 *M. minutus*, in 22 sesame fields that were separated by rivers, roads, and mountains. *M. musculus* were caught in 13 fields near the vicinity of human houses, whereas *M. minutus* were caught in 14 fields distant from human houses. Both species of wild mice shared a habitat in only 5 of 22 fields. Maps based on GPS coordinates were created using Quantum Geographical Information System, version 3.4.4 (http://qgis.org).

This study was reviewed and approved by the Institutional Animal Care and Use Committee of Hallym University (approval number Hallym2017-5). Wild mice, including *M. musculus* and *M. minutus*, were captured under piles of dry sesame plants in 22 sesame fields that were separated by rivers, roads, and mountains in Hongcheon and Chuncheon (Gangwon Province, South Korea) over the course of 2 years. The global positioning system (GPS) coordinates for the regions where the sesame fields were located are shown in Figure 1. All captured wild mice were immediately transported to the laboratory, and each wild mouse was transferred to a single disinfected cage. Fresh fecal samples were collected within 10 min and stored at 4°C. Subsequently, pathological signs or lesions and total length and body weight were investigated for each wild mouse. In *M. musculus*, the mean total lengths were 12.41 ± 0.43 (males) and 12.63 ± 0.56 cm (females), respectively, and the mean body weights were 9.61 ± 1.16 (males) and 9.93 ± 1.23 g (females), respectively. In *M. minutus*, the mean total lengths were 12.71 ± 0.2 (males) and 11.72 ± 0.95 cm (females), respectively, and the mean body weights were 7.44 ± 1.41 (males) and 8.38 ± 1.76 g (females), respectively.

Within 3 h after fecal sampling, we began isolating *C. jejuni* from the feces of the wild mice. Fecal samples collected from each wild mouse were homogenized in phosphate-buffered saline. The homogenized contents were directly spread onto modified charcoal–cefoperazone–deoxycholate agar plate (mCCDA; Oxoid, Ltd., Hampshire, United Kingdom) with a CCDA-selective supplement (Oxoid, Ltd., Hampshire, United Kingdom). Next, all plates were incubated at 42°C for 2 days under 83% N_2_, 7% CO_2_, 4% H_2_, and 6% O_2_ (Mace et al., 2015) in micro-aerobic jars using Anoxomat Mark II (MART Microbiology B.V., Lichtenvoorde, Netherlands). Next, colonies suspected to be *C. jejuni* were transferred to Müller–Hinton agar (Oxoid, Ltd., Hampshire, United Kingdom), and genomic DNA was extracted from each colony using the boiling method. Briefly, bacterial cells were suspended in 200 μL sterile distilled water, boiled for 10 min, and centrifuged at 13,000 × *g* for 3 min. The resulting supernatants were used as a template for polymerase chain reaction (PCR) experiments. *C. jejuni* was confirmed by running PCR experiments (Table 1; Wang et al., 2002). Then, to identify false negatives for *C. jejuni*, an isolation method with an enrichment process was applied. Briefly, the fecal samples that were negative for *C. jejuni* were enriched in Bolton broth (Oxoid, Ltd., Hampshire, United Kingdom) with a Bolton broth-selective supplement (Oxoid, Ltd., Hampshire, United Kingdom), and the enrichment broths were incubated at 42°C for 48 h under 83% N_2_, 7% CO_2_, 4% H_2_, and 6% O_2_ in micro-aerobic jars using Anoxomat Mark II (MART Microbiology B.V., Lichtenvoorde, Netherlands). Subsequently, the presence of *C. jejuni* in these samples was confirmed following the same procedure described above. Finally, the chi-square test was performed to compare the isolation rates of *C. jejuni* by the gender/age of the mice and the region/year in which mice were captured.

**TABLE 1:** Primes used for polymerase chain reaction (PCR) in this study.

	**Target microbe**	**Target**	**Size (bp)**	**Primer**	**Sequence (5′-3′)**	**References**
PCR for confirmation of	*C. jejuni*	*hipO*	323	F	ACTTCTTTATTGCTTGCTGC	Wang et al., 2002
*C. jejuni* colonies				R	GCCACAACAAGTAAAGAAGC	
	*Campylobacter* spp.	23S rRNA	650	F	TATACCGGTAAGGAGTGCTGGAG	Wang et al., 2002
				R	ATCAATTAACCTTCGAGCACCG	
PCR for identification of	*C. jejuni*	*flhB*	549	F	TGGCAGGCGAAGATCAAGAA	Koolman et al., 2015
virulence and survival-related				R	GCCAAGTAAGCTGTGCAACC	
genes of *C. jejuni* isolates		*virB11*	329	F	TCAGGTGGAACAGGAAGTGG	Koolman et al., 2015
				R	GCTTTGATCGCGTCTTCTGG	
		*hcp*	463	F	CAAGCGGTGCATCTACTGAA	Harrison et al., 2014
				R	TAAGCTTTGCCCTCTCTCCA	
		*cad*F	400	F	TTGAAGGTAATTTAGATATG	Konkel et al., 1999
				R	CTAATACCTAAAGTTGAAAC	
		*pld*A	913	F	AAGCTTATGCGTTTTT	Datta et al., 2003
				R	TATAAGGCTTTCTCCA	
		*iam*A	518	F	GCACAAAATATATCATTACAA	Müller et al., 2006
				R	TTCACGACTACTATGAGG	
		*cdt*B	376	F	GCTCCTACATCAACGCGAGA	Koolman et al., 2015
				R	ACTACTCCGCCTTTTACCGC	
		*crs*A	878	F	CACAGTCAGTGAAGGTGCTT	González-Hein et al., 2013
				R	ACTCGCACAATCGCTACTTC	
		*per*R	153	F	CCCTTCAATCTCTTTAGCGACG	An et al., 2018
				R	ATACCACCACATTTGGCGCA	
		*htr*A	130	F	CCATTGCGATATACCCAAACTT	Bui et al., 2012
				R	CTGGTTTCCAAGAGGGTGAT	
PCR for confirmation of	*C. jejuni*	*tet*O	559	F	GGCGTTTTGTTTATGTGCG	Gibreel et al., 2004
antibiotic resistance of				R	ATGGACAACCCGACAGAAGC	
*C. jejuni* isolates		gyr*A*		F	TTTTTAGCAAAGATTCTGAT	Zirnstein et al., 1999
			265	R1	CAAAGCATCATAAACTGCAA	
			368	R2	CAGTATAACGCATCGCAG	

### Multilocus Sequence Typing of Wild Mouse-Derived *C. jejuni*

Multilocus sequence typing (MLST) was performed on all *C. jejuni* isolates from wild mice, according to the PubMLST protocol^1^. Briefly, seven housekeeping genes (aspartase A, glutamine synthetase, citrate synthase, serine hydroxymethyl transferase, phosphoglucomutase, transketolase, and ATP synthase alpha subunit) were amplified and sequenced with primer sets described in the PubMLST protocol. Amplification and sequencing were performed using Lamp Taq DNA Polymerase (BIOFACT, South Korea) and an ABI 3730XL DNA analyzer (Applied Biosystems, United States), respectively. Furthermore, a minimum-spanning tree based on the allelic MLST profiles of the current study (ST-8388) and other sequence types (STs) registered in PubMLST (accessed on 20 November 2019, about 6,800 STs from *C. jejuni*, Supplementary Table 1) was generated using the PHYLOViZ software^2^ (Francisco et al., 2012).

### Profiling Virulence/Survival Factors and Antibiotic-Resistance Patterns of Wild Mouse-Derived *C. jejuni*

For all *C. jejuni* isolates, the presence and absence of virulence/survival-related genes were confirmed by PCR (Table 1; Konkel et al., 1999; Datta et al., 2003; Müller et al., 2006; Bui et al., 2012; González-Hein et al., 2013; Harrison et al., 2014; Koolman et al., 2015; An et al., 2018). These genes corresponded to three secretion systems (*flhB*, *vir*B11, and *hcp*), one adherence/colonization-related protein (*cad*F), two cell invasion-related proteins (*pld*A and *iam*A), one cytotoxin-related protein (*cdt*B), and three survival-related factors (*crs*A, *per*R, and *htr*A). The minimum inhibitory concentration (MIC; for erythromycin, chloramphenicol, ciprofloxacin, tetracycline, telithromycin, gentamicin, azithromycin, streptomycin, and nalidixic acid), disk diffusion (for ciprofloxacin, tetracycline, and nalidixic acid), and sequence-based tests (for *gyr*A and *tet*O genes) were conducted for all *C. jejuni* isolates to evaluate antibiotic resistance (Table 1; Zirnstein et al., 1999; Gibreel et al., 2004).

### Whole-Genome Sequencing of Representative Wild Mouse-Derived *C. jejuni* Strains

A representative strain of wild mouse-derived *C. jejuni* (strain SCJK2) was selected, and genomic DNA was extracted using the MG^TM^ Genomic DNA Purification Kit (Macrogen, South Korea). Whole-genome sequencing was performed using a Pacific Biosciences RS II sequencer (Pacific Biosciences, Menlo Park, CA, United States). *De novo* microbial genome assemblies were carried out using Hierarchical Genome Assembly Process, version 3.0 (Chin et al., 2013), and all contigs were circularized using Circlator 1.4.0 (Sanger Institute, United Kingdom). The NCBI prokaryotic genome annotation pipeline^3^ and the EzBioCloud genome database^4^ were used for gene annotation. Functional classification was performed using the EggNOG (Powell et al., 2014), SwissProt (Pundir et al., 2015), KEGG (Kanehisa et al., 2015), and SEED (Overbeek et al., 2005) databases. Antibiotic resistance- and virulence-related genes of wild mouse-derived *C. jejuni* (strain SCJK2) sequence were analyzed using the comprehensive antibiotic resistance database (CARD) (Jia et al., 2016) and virulence factors of pathogenic bacteria database (VFDB) (Chen et al., 2015), respectively.

### Comparative-Genomic Analysis of Wild Mouse-Derived *C. jejuni* With Previously Reported Isolates

For comparative genomic analysis, 174 complete genome sequences of *C. jejuni* from various sources, including humans, poultry, bovines, and sheep, as well as environmental isolates (Supplementary Table 2), were downloaded from the NCBI Genome database^5^. For phylogenetic characterization of the wild mouse-derived *C. jejuni* (strain SCJK2) sequence with other sequences obtained from the NCBI Genome database, functional annotation of all 175 genomes was performed using Prokka (Seemann, 2014), and then core genes (genes possessed by >95% genomes) and accessory genes (genes possessed by <95% strains) were identified using Roary (Page et al., 2015). Multiple alignments were performed on core genes and approximately maximum-likelihood phylogenetic tree was constructed with generalized time reversible models using PRANK and Fast Tree (Price et al., 2010; Löytynoja, 2014). The phylogenetic tree was generated using the iTOl^6^.

## Results

### Habitat of Each Wild Mouse Species

We captured 115 wild mice, including 49 *M. musculus* (20 male and 29 female) and 66 *M. minutus* (40 male and 26 female) in 22 sesame fields; *M. musculus* were captured in 13 out of 22 sesame fields and *M. minutus* were captured in 14 out of 22 sesame fields (Figure 1 and Table 2). *M. musculus* were typically caught in the vicinity of human houses, whereas *M. minutus* were caught in remote areas of human houses, or near mountains or rivers. Both species of wild mice shared a habitat in only 5 of 22 sesame fields (fields 02, 03, 05, 09, and 13). Pathological signs or lesions were not observed in any of the captured wild mice.

**TABLE 2:** Prevalence of *Campylobacter jejuni* and the *vir*B11 gene from *C jejuni* in the different wild mouse species (*Mus musculus* and *Micromys minutus*).

**Species**	***Mus musculus***		**Species**	***Micromys minutus***		
**Field**	**Number of mouse (female/male)**	**Number of *C. jejuni* positive (female/male)**	**Field**	**Number of mouse (female/male)**	**Number of *C. jejuni* positive (female/male)**	**Number of *vir*B11 positive isolates**
01	12 (7/5)	–	02	3 (2/1)	3 (2/1)	3 (100%)
02	2 (2/0)	–	03	9 (2/7)	6 (0/6)	0 (0%)
03	8 (7/1)	–	05	1 (1/0)	–	–
04	1 (1/0)	–	09	3 (1/2)	1(0/1)	0 (0%)
05	3 (0/3)	–	13	9 (6/3)	5 (4/1)	5 (100%)
06	1 (0/1)	–	14	5 (3/2)	3 (1/2)	0 (0%)
07	3 (1/2)	–	15	10 (2/8)	5 (0/5)	0 (0%)
08	1 (0/1)	–	16	3 (2/1)	2 (1/1)	0 (0%)
09	1 (0/1)	–	17	12 (3/9)	10 (3/7)	10 (100%)
10	7 (5/2)	–	18	1 (0/1)	1 (0/1)	0 (0%)
11	4 (3/1)	–	19	1 (1/0)	–	–
12	3 (1/2)	–	20	3 (2/1)	3 (2/1)	0 (0%)
13	3 (2/1)	–	21	2 (0/2)	1 (0/1)	0 (0%)
			22	4 (1/3)	2 (0/2)	2 (100%)
Total	49 (29/20)	0 (0%)	Total	66 (26/40)	42 (63.6%)	20 (47.62%)

### Prevalence and Clonal Distribution of *C. jejuni* in Wild Mice

*Campylobacter jejuni* was not isolated from any of the 49 *M. musculus* (0%), whereas *C. jejuni* was isolated from 42 of 66 *M. minutus* (63.6%). *C. jejuni* was isolated in 12 out of 14 sesame fields where *M. minutus* were captured; only one *M. minutus* mouse each was captured from field 05 and from field 19, where *C. jejuni* was not isolated. In addition, even in cases where the two species shared the same field, *C. jejuni* was isolated only from *M. minutus* (Table 2). No difference was observed in the prevalence of *C. jejuni* in *M. minutus* based on the gender/age of the wild mice or the region/year in which wild mice were captured.

Multilocus sequence typing analysis for all isolates revealed that the sequences of seven housekeeping genes (*asp*A, *gln*A, *glt*A, *gly*A, *pgm*, *tkt*, and *unc*A) were identical, regardless of various factors (gender, age, region, and year). In other words, only one ST was found among all 42 *C. jejuni* isolates from *M. minutus*. All sequences were submitted to PubMLST and a new ST, ST-8388 (*asp*A: 7, *gln*A: 618, *glt*A: 303, *gly*A: 688, *pgm*: 823, *tkt*: 645, and *unc*A: 6), was determined.

### Virulence/Survival Factors Profile and Antibiotic-Resistance Patterns of *C. jejuni* in Wild Mice

Among 10 virulence- and survival-related genes, *flh*B, *cad*F, *pld*A, *iam*A, *cdt*B, *crs*A, *per*R, and *htr*A were present in all (100%) *C. jejuni* isolates. However, *hcp* was not detected in any isolates. In addition, *vir*B11 was detected in 20/42 (47.6%) of isolates with a similar geographical distribution (Table 2). Specifically, all 20 isolates found in fields 02 (3 isolates), 13 (5 isolates), 17 (10 isolates), and 22 (2 isolates) were positive for *vir*B11, whereas 22 isolates from other fields (fields 03, 05, 09, 14, 15, 16, 18, 19, 20, and 21) were negative for this gene. Wild mice were caught in different 2 years from fields 03 and 13, but an identical pattern was observed. In an antibiotic-resistance test, all *C. jejuni* isolates were susceptible to all tested antibiotics, regardless of testing method used (MIC, disk diffusion, or a sequence-based method).

### Complete Genome Sequence and Genomic Characterization of Wild Mouse-Derived *C. jejuni*

One representative *C. jejuni* strain (SCJK2) was selected for sequencing, since all 42 isolates from *M. minutus* were presumed to be genetically identical, according to the MLST results. Based on the whole-genome sequence of *M. minutus*-derived *C. jejuni* (strain SCJK2), the complete genome has a total length of 1,859,287 bp and consists of three contigs (one chromosome and two plasmids) with an average GC content of 30.15%. The genome harbors 1,781 coding genes (chromosome: 1,642 genes; plasmid 1: 93 genes; plasmid 2: 46 genes), 44 tRNA, 9 rRNA, and 3 ncRNA (Table 3). Among these, 1,278 coding genes are assigned to EggNog/COG categories. The complete genome sequence of *M. minutus*-derived *C. jejuni* (strain SCJK2) was deposited in GenBank (accession numbers CP038862–CP038864). In virulence-factor analysis based on VFDB, the *M. minutus*-derived *C. jejuni* genome (strain SCJK2) contains most virulence-related genes associated with adherence (*cad*F, *jlp*A, *por*A, and *peb*A), colonization (capsule biosynthesis and transport-related genes), glycosylation (N-linked protein and O-linked flagella glycosylation-related genes), immune evasion (LOS-related genes), invasion (*cia*B), motility (flagella-related genes), the secretion system (type IV secretion system-related genes), and toxins (*cdt*A/B/C). Antibiotic-resistance analysis indicated that the genome did not contain antibiotic-resistance genes which correspond to genes registered in CARD.

**TABLE 3:** Genomic features of *M. minutus*-derived *C. jejuni* (str SCJK2).

**Feature**	**Chromosome**	**Plasmid 1**	**Plasmid 2**	**Total**
Length (bp)	1,737,053	86,091	36,143	1,859,287
GC contents (%)	30.4	27.9	25.9	30.2
Number of coding genes	1,642	93	46	1,781
Number of tRNA	44	0	0	44
Number of rRNA	9	0	0	9
Number of ncRNA	3	0	0	3

### Genetic Relatedness Between Wild Mouse-Derived *C. jejuni* and *C. jejuni* Derived From Other Sources, Based on MLST and Comparative-Genomic Analysis

**FIGURE 2:**
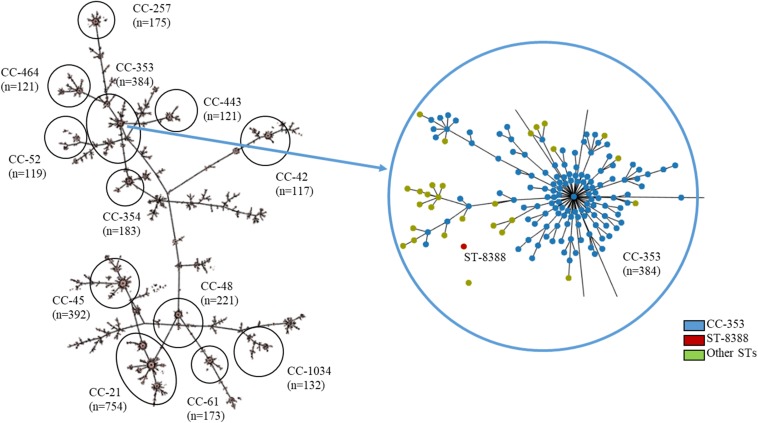
MLST results showing the genetic relatedness between *C. jejuni* derived from *Micromys minutus* and those derived from other sources. Minimum-spanning tree based on the allelic profile of MLST, including ST-8388 isolated in this study and other STs registered in PubMLST (accessed on 20 November 2019, about 6,800 STs, https://pubmlst.org/). Cut-off value of the tree was set to allele differences of 5.0. The distance between *M. minutus*-derived *C. jejuni* (ST-8388) and the nearest ST (ST-353) was 5.0. This figure was generated using the Phyloviz software (http://www.phyloviz.net/).

**FIGURE 3:**
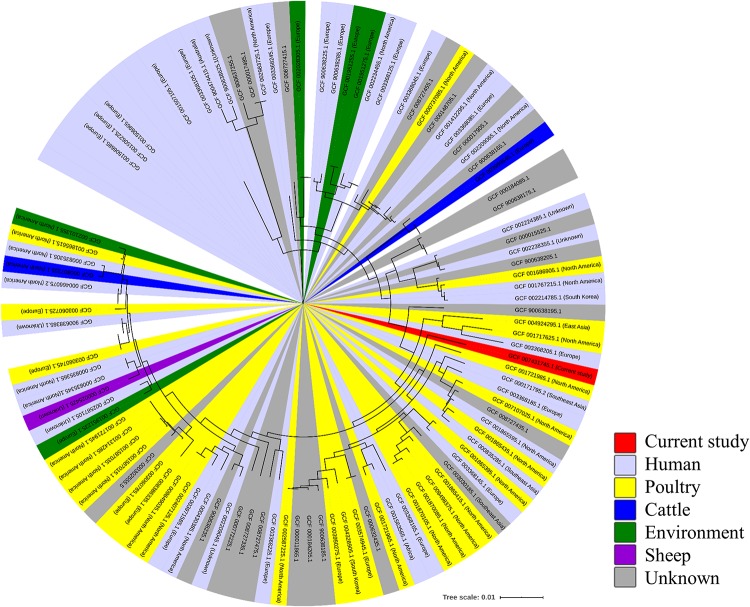
Comparative-genomic analysis of *Micromys minutus*-derived *C. jejuni* sequences (strain SCJK2) with 174 other *C. jejuni* sequences. An approximately maximum-likelihood phylogenetic tree based on multiple alignment of core genes. Nodes lower than the cut-off value (branch length of 0.00005) were collapsed. The *M. minutus*-derived *C. jejuni* (strain SCJK2) sequence was not deeply clustered with other sequences, including GCF 002214785.1 and GCF 004328905.1, which were isolated from South Korea. In addition, the branch length (0.009) from the closet bootstrap in the tree of this strain was much longer than that of most other strains. This figure was generated using the iTOl (https://itol.embl.de/).

A minimum-spanning tree was constructed based on MLST and was used to assess allelic differences between sequences of *M. minutus*-derived *C. jejuni* and previously reported sequences (Figure 2). In the case of *C. jejuni* from *M. minutus*, the allelic difference to the nearest ST was 5.0, indicating a low level of genetic relatedness with *C. jejuni* from other sources, such as humans, livestock, and the environment. The complete genome of *M. minutus*-derived *C. jejuni* (strain SCJK2) was compared with 174 other *C. jejuni* genome sequences from different sources (Figure 3). Among 1,203 core genes of 175 genomes, *M. minutus*-derived *C. jejuni* (strain SCJK2) did not harbor 12 genes, including *aer*_1 (aerotaxis receptor), *gln*M (putative glutamine ABC transporter permease protein), *che*Y (chemotaxis protein), *tyr*S (tyrosine-tRNA ligase), and *peb*1A (major cell-binding factor). In the approximately maximum-likelihood phylogenetic tree, this genome sequence formed clades with other sequences. However, unlike most other sequences that were clustered by isolated country or source, this genome was not deeply clustered with other sequences, including GCF 002214785.1 and GCF 004328905.1, which were isolated from South Korea. In addition, the branch length from the closet bootstrap (0.009) in the tree of this strain was much longer than that of most other strains.

## Discussion

This is the first study to identify and characterize *C. jejuni* from wild mice, in addition to analyzing the genetic relatedness of wild mouse-derived strains and those derived from other sources, including humans and livestock. Wildlife animals cause many infectious diseases in humans. In particular, wild mice are known to carry various pathogens that cause diseases such as Hantavirus pulmonary syndrome 1, plague, and typhus (Kruse et al., 2004; Wolfe et al., 2007). It is likely that wild mice may be reservoirs for *C. jejuni* infection for humans and livestock, but this has not been clearly demonstrated (Meerburg and Kijlstra, 2007). Therefore, the objectives of this study were to determine whether wild mice are a potential reservoir of *C. jejuni* and whether they are involved in the transmission cycle of *C. jejuni* in human infections. To accomplish this, we applied traditional research approaches, such as profiling of virulence-/survival-related genes and evaluating antibiotic resistance, in addition to performing MLST analysis. Furthermore, we utilized a whole-genome sequencing/comparative-genomic analysis approach for in-depth characterization of *C. jejuni* from wild mice and for in-depth analysis of the genetic relatedness between wild mouse-derived *C. jejuni* and *C. jejuni* derived from previously reported sources.

We found that most *M. minutus* harbored *C. jejuni* in their gastro-intestinal tracts, regardless of various factors, such as the gender/age of the mice and the region/year in which the mice were captured. The prevalence of *C. jejuni* in *M. minutus* was 63.6% on an individual basis (42 of 66 *M. minutus*) and 85.7% on a regional basis (12 of 14 sesame fields). Furthermore, we did not observe any clinical symptoms or lesions in any *M. minutus* from which *C. jejuni* was isolated. Generally, in humans, *C. jejuni* is pathogenic and causes campylobacteriosis, which can result in gastro-intestinal and neurological diseases (Nachamkin et al., 1998). However, in poultry, *C. jejuni* generally exists as a commensal flora in the gastro-intestinal tract and does not cause any clinical symptoms (Silva et al., 2011). Furthermore, its prevalence in chickens exceeds 50% (Skarp et al., 2016; Kim et al., 2019). Therefore, the high prevalence of *C. jejuni* in wild mice combined with the absence of any clinical symptoms suggest that *C. jejuni* could be a member of the normal gut flora in wild mice, similar to the situation with poultry.

We performed MLST, the most commonly used epidemiological tool for *C. jejuni* (Dingle et al., 2001), on all isolates to investigate the origin of *C. jejuni* in wild mice, in addition to the genetic relationship among isolates. Surprisingly, all 42 isolates from wild mice were from a single clone *C. jejuni* (ST-8388), although these *M. minutus* were captured from different habitats during different years, and were not all of the same gender. Moreover, this MLST type had not been previously reported. It is generally known that the genetic diversity of *C. jejuni* is higher than that of other microorganisms due to frequent genetic exchange (Young et al., 2007). Therefore, a variety of genetically different *C. jejuni* clones is distributed worldwide (Dingle et al., 2002), and more than 6,800 *C. jejuni* MLST types are registered in PubMLST (accessed on 20 November 2019). Previous studies on livestock, including cattle, sheep, pigs, and poultry have indicated that, though the frequency of a specific sequence is often host-dependent, diverse genotypes of *C. jejuni* strains can be distributed among the same hosts and individuals (Colles et al., 2009; Ogden et al., 2009). For example, in cattle or poultry, various clones of *C. jejuni* may be present, even if the experiment was carried out on the same farm (Behringer et al., 2011; An et al., 2018). Despite the previously determined genetic diversity of *C. jejuni*, the absence of genetic heterogeneity among all isolates in this study suggests that *C. jejuni* from wild mice may have adapted to the gastrointestinal tract (GI) of *M. minutus* and that all isolates were genetically similar to each other, a result that has not been seen with other *C. jejuni* hosts. In addition, *M. minutus*-derived *C. jejuni* formed separate clusters from other host-derived *C. jejuni* in the MLST analysis, suggesting that the origin of *C. jejuni* in wild mice might differ from that of other hosts, including livestock and humans.

In this study, none of the *C. jejuni* isolates from wild mice exhibited resistance to any antibiotics either phenotypically or genetically. Antibiotic-resistance patterns are generally used as an indicator of exposure histories for previous antibiotic use. Recently, due to the increased use of antibiotics for therapeutic or sub-therapeutic purposes in humans and livestock, a greater number of *C. jejuni* antibiotic-resistant strains have been found worldwide, especially those resistant to fluoroquinolones and tetracycline (Alfredson and Korolik, 2007; Luangtongkum et al., 2009). Antibiotic resistance in *C. jejuni* has been found to be especially severe in Korea. According to statistics provided by the Animal and Plant Quarantine Agency of Korea^7^ (accessed on May 2019) over the last 2 years, the antibiotic-resistance rates of poultry-derived *C. jejuni* against ciprofloxacin, nalidixic acid, and tetracycline were 97.4, 94.7, and 63.2%, respectively. In addition, almost all *C. jejuni* isolates from humans and livestock in Korea were resistant to at least one antibiotic (Kim et al., 2016; Chon et al., 2018). However, in this study, the antibiotic-resistance patterns of *C. jejuni* from wild mice deviated from this pattern. These results might provide evidence that wild mice were not previously exposed to antibiotics. Furthermore, considering that *C. jejuni* acquires antibiotic resistance by taking up foreign DNA, which makes antibiotic resistance easily transmitted between strains (Bae et al., 2014), *C. jejuni* from wild mice most likely had not been transmitted from other sources, such as livestock or humans that were frequently exposed to antibiotics.

Furthermore, in this study, *C. jejuni* from wild mice also had unique virulence/survival profiles compared to those of *C. jejuni* derived from other sources. Because of the high genetic diversity of *C. jejuni* mentioned above, differences in the virulence/survival profiles of *C. jejuni* have been observed in most previous studies for the same host, and even in studies of livestock in the same farm (Young et al., 2007; An et al., 2018). However, the presence or absence of virulence/survival genes in all isolates from this study was similar, except for the *vir*B11 gene. This similarity might be related to the distribution of single *C. jejuni* clone among the *M. minutus*, as mentioned in the section “Genetic Relatedness Between Wild Mouse-Derived *C. jejuni* and *C. jejuni* Derived From Other Sources, Based on MLST and Comparative-Genomic Analysis.” The presence/absence of the *vir*B11 gene was not the same for each isolate. However, if a single *C. jejuni* isolate from wild mice captured in one sesame field harbored this gene, then all isolates from wild mice captured in that field harbored the gene, even though the mouse were captured under different piles of dry sesame plants. Similarly, if a single *C. jejuni* isolate from wild mice captured in one sesame field did not harbor this gene, then no isolates from wild mice captured in that field harbored the gene (Table 2). *Vir*B11, which is present on the pVir plasmid encoding a type-IV secretion system, is involved in horizontal gene transfer and is easily transferred between strains (Juhas et al., 2008); in the case of livestock, its distribution tendency has been found to be similar for the same farm (An et al., 2018). Therefore, the distribution of the *vir*B11 gene in this study suggests that horizontal transmission of *C. jejuni* may occur frequently among *M. minutus* that share the same habitat. Alternatively, vertical transmission of *C. jejuni* harboring the *vir*B11 gene may occur continuously, since wild mice captured in the same sesame field could all be family members.

To investigate the possibility of *C. jejuni* transmission from wild mice to humans, we investigated the habitat of wild mice (Figure 1). Two possible transmission routes can be considered for an event from wild mice to humans: either direct transmission through contact with the wild mice, or indirect transmission through the environment or livestock. Both routes involve sharing of a neighboring environment between humans and wild mice. *M. musculus* and *M. minutus* are widely distributed throughout Asia and Europe (Miyashita et al., 1985; Jing et al., 2015), and in particular, these two species are common in the South Korea (Kim et al., 2013). *M. musculus*, which has been domesticated as a laboratory mouse or pet, have been found in close proximity to commercial structures and have come into contact with humans (Panti-May et al., 2012). In contrast, *M. minutus* live in open communities, including mountains and agricultural fields, and have a unique nesting behavior, where small shelter nests are built off the ground (Jing et al., 2015). In this study, even though two wild mice species were caught in sesame fields, *M. musculus* were typically caught in the vicinity of human houses, indicating that *M. musculus* most likely have more contact with humans. However, *M. minutus* were caught in remote areas of human houses, which reduced the possibility of human contact. Therefore, since *C. jejuni* was isolated only from *M. minutus*, and there was a low chance of contact between *M. minutus* and humans/livestock, the probability of *C. jejuni* transmission from wild mouse to humans can be assumed to be relatively low.

Furthermore, to identify genetic traces of *M. minutus*-derived *C. jejuni* in other hosts, we compared the genetic relatedness of *C. jejuni* from *M. minutus* and other sources previously reported in the PubMLST and NCBI Genome databases, using MLST and comparative-genomic analysis. The relatively large distance of *C. jejuni* wild mouse isolates from other STs (i.e., from a different host or source) in the MST based on MLST allelic profiles suggests that *M. minutus*-derived *C. jejuni* is a novel strain that is not genetically closely related to *C. jejuni* derived from other sources. In the comparative genomic analysis conducted in this study, we did not find great genetic relatedness between *M. minutus*-derived *C. jejuni* and those derived from other sources (humans, livestock, environmental sources, and other wildlife). These results also indicate that *C. jejuni* derived from *M. minutus* might be a novel strain and has a different ancestral lineage from those derived from other hosts. Furthermore, there are no evidence for a genetic trace that could indicate a role for wild mice in the transmission of *C. jejuni* to other hosts.

## Conclusion

In conclusion, based on the absence of clinical signs in wild mice harboring *C. jejuni*, in addition to its high prevalence, single clonal distribution, and lack of resistance to any tested antibiotics, we hypothesize that *C. jejuni* most likely forms a normal component of the gut flora of *M. minutus* and all *C. jejuni* isolates are adapted to the GI tract of *M. minutus*. In addition, the probability of *C. jejuni* transmission from *M. minutus* to humans is relatively low because *M. minutus* and other hosts did not share neighboring environments and genetic relatedness was not observed between *C. jejuni* derived from *M. minutus* and other sources. In summary, *M. minutus* might be a potential reservoir of *C. jejuni*, a novel strain that is genetically different from those of other sources, but is most likely not involved in *C. jejuni* transmission to other hosts, including humans and livestock.

## Data Availability Statement

The datasets (genome sequence of *C. jejuni* strain SCJK2) for this study were deposited in GenBank under accession numbers CP038862–CP038864.

## Ethics Statement

The animal study was reviewed and approved by the Institutional Animal Care and Use Committee (IACUC) of Hallym University approval (Hallym2017-5).

## Author Contributions

SC and JGS conceived the study. J-HG, S-HM, J-UA, WK, SL, and HS performed the sampling and experiments. JKS prepared and reviewed the manuscript. JK made a great contribution to the experiments and preparing the manuscript. All authors have approved the manuscript.

## Conflict of Interest

The authors declare that the research was conducted in the absence of any commercial or financial relationships that could be construed as a potential conflict of interest.

## Footnotes

1 https://pubmlst.org/

2 http://www.phyloviz.net/

3 https://www.ncbi.nlm.nih.gov/genome/annotation_prok/

4 https://www.ezbiocloud.net/

5 https://www.ncbi.nlm.nih.gov/genome/

6 https://itol.embl.de/

7 http://www.qia.go.kr/
