# Supplementary material for: The Wild Mouse (Micromys minutus): Reservoir of a Novel Campylobacter jejuni Strain
Source: Front Microbiol. 2020 Jan 14;10:3066. doi: 10.3389/fmicb.2019.03066 (PMC6971111; doi:10.3389/fmicb.2019.03066)
Supplement: Supplementary file 2 [file Data_Sheet_1.docx]

Supplementary Material

# Supplementary Tables

**Supplementary Table 1.** The 6,813 different sequence types (ST) registered in PubMLST used to create the minimum spanning tree.

**Supplementary Table 2.** The 174 complete genome sequences of *C. jejuni* obtained from the NCBI database used for comparative genomic analysis.

| **No.** | **Assembly ID** | **Source** | **Nation** | **MLST**  **sequence type** | **MLST**  **clonal complex** |
| --- | --- | --- | --- | --- | --- |
| **1** | GCF_001506965.1 | Human | Europe | ST-677 | ST-677 complex |
| **2** | GCF_003368105.1 | Human | Europe | ST-5673 | - |
| **3** | GCF_003368245.1 | Human | Europe | ST-508 | ST-508 complex |
| **4** | GCF_900638225.1 | Human | Europe | ST-45 | ST-45 complex |
| **5** | GCF_900638285.1 | Human | Europe | ST-45 | ST-45 complex |
| **6** | GCF_003368125.1 | Human | Europe | ST-45 | ST-45 complex |
| **7** | GCF_003368045.1 | Human | Europe | ST-137 | ST-45 complex |
| **8** | GCF_003368085.1 | Human | Europe | ST-230 | ST-45 complex |
| **9** | GCF_003368205.1 | Human | Europe | ST-1332 | ST-1332 complex |
| **10** | GCF_003368185.1 | Human | Europe | ST-3140 | ST-464 complex |
| **11** | GCF_003368145.1 | Human | Europe | ST-52 | ST-52 complex |
| **12** | GCF_003368165.1 | Human | Europe | ST-5671 | ST-443 complex |
| **13** | GCF_003368225.1 | Human | Europe | ST-1972 | - |
| **14** | GCF_003971585.1 | Human | Europe | ST-221 | ST-206 complex |
| **15** | GCF_001506685.1 | Human | Europe | ST-677 | ST-677 complex |
| **16** | GCF_001506225.1 | Human | Europe | ST-677 | ST-677 complex |
| **17** | GCF_001507105.1 | Human | Europe | ST-794 | ST-677 complex |
| **18** | GCF_002983725.1 | Human | North America | ST-1845 | - |
| **19** | GCF_002234455.1 | Human | North America | ST-45 | ST-45 complex |
| **20** | GCF_001412295.1 | Human | North America | ST-137 | ST-45 complex |
| **21** | GCF_002209065.1 | Human | North America | ST-583 | ST-45 complex |
| **22** | GCF_001767215.1 | Human | North America | ST-22 | ST-22 complex |
| **23** | GCF_001865595.1 | Human | North America | ST-353 | ST-353 complex |
| **24** | GCF_000430385.1 | Human | North America | ST-1460 | ST-48 complex |
| **25** | GCF_000835345.1 | Human | North America | ST-8 | ST-8 complex |
| **26** | GCF_000835365.1 | Human | North America | ST-8 | ST-8 complex |
| **27** | GCF_000466075.2 | Human | North America | ST-21 | ST-21 complex |
| **28** | GCF_000835305.1 | Human | North America | ST-806 | ST-21 complex |
| **29** | GCF_000171795.2 | Human | Southeast Asia | ST-1919 | ST-52 complex |
| **30** | GCF_000835285.1 | Human | Southeast Asia | ST-2132 | ST-353 complex |
| **31** | GCF_003030185.1 | Human | Southeast Asia | ST-6238 | ST-607 complex |
| **32** | GCF_001563565.1 | Human | Africa | ST-1471 | ST-460 complex |
| **33** | GCF_900474415.1 | Human | Australia | ST-8767 | - |
| **34** | GCF_002214785.1 | Human | South Korea | ST-22 | ST-22 complex |
| **35** | GCF_003574945.1 | Poultry | Europe | ST-356 | ST-353 complex |
| **36** | GCF_003950275.1 | Poultry | Europe | ST-354 | ST-354 complex |
| **37** | GCF_008386335.1 | Poultry | Europe | ST-44 | ST-21 complex |
| **38** | GCF_003060785.1 | Poultry | Europe | ST-50 | ST-21 complex |
| **39** | GCF_003060725.1 | Poultry | Europe | ST-53 | ST-21 complex |
| **40** | GCF_003060745.1 | Poultry | Europe | ST-21 | ST-21 complex |
| **41** | GCF_000737085.1 | Poultry | North America | ST-8785 | ST-45 complex |
| **42** | GCF_001686905.1 | Poultry | North America | ST-22 | ST-22 complex |
| **43** | GCF_001717625.1 | Poultry | North America | ST-1839 | - |
| **44** | GCF_001721985.1 | Poultry | North America | ST-1911 | - |
| **45** | GCF_007107025.1 | Poultry | North America | ST-353 | ST-353 complex |
| **46** | GCF_001865435.1 | Poultry | North America | ST-462 | ST-353 complex |
| **47** | GCF_001865395.1 | Poultry | North America | ST-3647 | ST-52 complex |
| **48** | GCF_001865415.1 | Poultry | North America | ST-607 | ST-607 complex |
| **49** | GCF_009498375.1 | Poultry | North America | ST-1212 | ST-607 complex |
| **50** | GCF_001870085.1 | Poultry | North America | ST-1212 | ST-607 complex |
| **51** | GCF_001870105.1 | Poultry | North America | ST-1212 | ST-607 complex |
| **52** | GCF_001721965.1 | Poultry | North America | ST-460 | ST-460 complex |
| **53** | GCF_002587225.1 | Poultry | North America | ST-354 | ST-354 complex |
| **54** | GCF_002407125.1 | Poultry | North America | ST-222 | ST-206 complex |
| **55** | GCF_009649035.1 | Poultry | North America | ST-222 | ST-206 complex |
| **56** | GCF_001587015.1 | Poultry | North America | ST-50 | ST-21 complex |
| **57** | GCF_001587035.1 | Poultry | North America | ST-50 | ST-21 complex |
| **58** | GCF_001314285.1 | Poultry | North America | ST-50 | ST-21 complex |
| **59** | GCF_001721945.1 | Poultry | North America | ST-8789 | ST-21 complex |
| **60** | GCF_001865615.1 | Poultry | North America | ST-21 | ST-21 complex |
| **61** | GCF_004924295.1 | Poultry | East Asia | ST-9042 |  |
| **62** | GCF_004328905.1 | Poultry | South Korea | ST-6849 | ST-354 complex |
| **63** | GCF_003999645.1 | Cattle | Europe | ST-403 | ST-403 complex |
| **64** | GCF_000807355.1 | Cattle | North America | ST-806 | ST-21 complex |
| **65** | GCF_000025425.1 | Sheep | Unknown | ST-8 | ST-21 complex |
| **66** | GCF_002028305.1 | Environment | Europe | ST-5843 | - |
| **67** | GCF_001951255.1 | Environment | Europe | ST-45 | ST-45 complex |
| **68** | GCF_001951275.1 | Environment | Europe | ST-45 | ST-45 complex |
| **69** | GCF_001951235.1 | Environment | Europe | ST-861 | ST-21 complex |
| **70** | GCF_002101355.1 | Environment | North America | ST-982 | ST-21 complex |
| **71** | GCF_900638625.1 | Human | Unknown | ST-7665 | - |
| **72** | GCF_002224385.1 | Human | Unknown | ST-604 | ST-42 complex |
| **73** | GCF_002238355.1 | Human | Unknown | ST-604 | ST-42 complex |
| **74** | GCF_002209045.1 | Human | Unknown | ST-48 | ST-48 complex |
| **75** | GCF_002587105.1 | Human | Unknown | ST-883 | ST-21 complex |
| **76** | GCF_900638365.1 | Human | Unknown | ST-43 | ST-21 complex |
| **77** | GCF_900637255.1 | Unknown | Unknown | ST-62 | - |
| **78** | GCF_000017485.1 | Unknown | Unknown | ST-1845 | - |
| **79** | GCF_008727415.1 | Unknown | Unknown | ST-5453 | ST-179 complex |
| **80** | GCF_008727455.1 | Unknown | Unknown | ST-2109 | ST-45 complex |
| **81** | GCF_000148705.1 | Unknown | Unknown | ST-137 | ST-45 complex |
| **82** | GCF_000017905.1 | Unknown | Unknown | ST-267 | ST-283 complex |
| **83** | GCF_900638165.1 | Unknown | Unknown | ST-403 | ST-403 complex |
| **84** | GCF_000184085.1 | Unknown | Unknown | ST-2993 | ST-362 complex |
| **85** | GCF_900638175.1 | Unknown | Unknown | ST-362 | ST-362 complex |
| **86** | GCF_000015525.1 | Unknown | Unknown | ST-604 | ST-42 complex |
| **87** | GCF_900638205.1 | Unknown | Unknown | ST-22 | ST-22 complex |
| **88** | GCF_900638195.1 | Unknown | Unknown | ST-61 | ST-61 complex |
| **89** | GCF_008727435.1 | Unknown | Unknown | Not assigned | - |
| **90** | GCF_000632435.1 | Unknown | Unknown | ST-356 | ST-353 complex |
| **91** | GCF_900638185.1 | Unknown | Unknown | ST-354 | ST-354 complex |
| **92** | GCF_000184205.1 | Unknown | Unknown | ST-354 | ST-354 complex |
| **93** | GCF_000011865.1 | Unknown | Unknown | ST-354 | ST-354 complex |
| **94** | GCF_008727475.1 | Unknown | Unknown | Not assigned | - |
| **95** | GCF_008727335.1 | Unknown | Unknown | ST-441 | - |
| **96** | GCF_000772225.1 | Unknown | Unknown | ST-3644 | - |
| **97** | GCF_900638235.1 | Unknown | Unknown | ST-48 | ST-48 complex |
| **98** | GCF_000302555.5 | Unknown | Unknown | ST-50 | ST-21 complex |
| **99** | GCF_001506425.1 | Human | Europe | ST-677 | ST-677 complex |
| **100** | GCF_001506565.1 | Human | Europe | ST-677 | ST-677 complex |
| **101** | GCF_001506445.1 | Human | Europe | ST-677 | ST-677 complex |
| **102** | GCF_001506985.1 | Human | Europe | ST-677 | ST-677 complex |
| **103** | GCF_001506385.1 | Human | Europe | ST-677 | ST-677 complex |
| **104** | GCF_001506505.1 | Human | Europe | ST-677 | ST-677 complex |
| **105** | GCF_001506825.1 | Human | Europe | ST-677 | ST-677 complex |
| **106** | GCF_001506625.1 | Human | Europe | ST-677 | ST-677 complex |
| **107** | GCF_001506905.1 | Human | Europe | ST-677 | ST-677 complex |
| **108** | GCF_001506745.1 | Human | Europe | ST-677 | ST-677 complex |
| **109** | GCF_001506525.1 | Human | Europe | ST-677 | ST-677 complex |
| **110** | GCF_001507085.1 | Human | Europe | ST-677 | ST-677 complex |
| **111** | GCF_001506725.1 | Human | Europe | ST-677 | ST-677 complex |
| **112** | GCF_001507225.1 | Human | Europe | ST-677 | ST-677 complex |
| **113** | GCF_001506285.1 | Human | Europe | ST-677 | ST-677 complex |
| **114** | GCF_001506845.1 | Human | Europe | ST-677 | ST-677 complex |
| **115** | GCF_001507025.1 | Human | Europe | ST-677 | ST-677 complex |
| **116** | GCF_001507185.1 | Human | Europe | ST-677 | ST-677 complex |
| **117** | GCF_001506945.1 | Human | Europe | ST-677 | ST-677 complex |
| **118** | GCF_001507245.1 | Human | Europe | ST-677 | ST-677 complex |
| **119** | GCF_001507125.1 | Human | Europe | ST-677 | ST-677 complex |
| **120** | GCF_001506305.1 | Human | Europe | ST-677 | ST-677 complex |
| **121** | GCF_001506205.1 | Human | Europe | ST-677 | ST-677 complex |
| **122** | GCF_001506345.1 | Human | Europe | ST-677 | ST-677 complex |
| **123** | GCF_001506665.1 | Human | Europe | ST-677 | ST-677 complex |
| **124** | GCF_001507145.1 | Human | Europe | ST-677 | ST-677 complex |
| **125** | GCF_001506885.1 | Human | Europe | ST-677 | ST-677 complex |
| **126** | GCF_001507165.1 | Human | Europe | ST-677 | ST-677 complex |
| **127** | GCF_001506485.1 | Human | Europe | ST-677 | ST-677 complex |
| **128** | GCF_001506545.1 | Human | Europe | ST-677 | ST-677 complex |
| **129** | GCF_001506465.1 | Human | Europe | ST-677 | ST-677 complex |
| **130** | GCF_001506185.1 | Human | Europe | ST-677 | ST-677 complex |
| **131** | GCF_001506705.1 | Human | Europe | ST-677 | ST-677 complex |
| **132** | GCF_001507045.1 | Human | Europe | ST-677 | ST-677 complex |
| **133** | GCF_001506365.1 | Human | Europe | ST-677 | ST-677 complex |
| **134** | GCF_001507005.1 | Human | Europe | ST-677 | ST-677 complex |
| **135** | GCF_001506805.1 | Human | Europe | ST-677 | ST-677 complex |
| **136** | GCF_001506245.1 | Human | Europe | ST-677 | ST-677 complex |
| **137** | GCF_001507065.1 | Human | Europe | ST-677 | ST-677 complex |
| **138** | GCF_001506605.1 | Human | Europe | ST-794 | ST-677 complex |
| **139** | GCF_001507205.1 | Human | Europe | ST-794 | ST-677 complex |
| **140** | GCF_001506405.1 | Human | Europe | ST-794 | ST-677 complex |
| **141** | GCF_001506865.1 | Human | Europe | ST-794 | ST-677 complex |
| **142** | GCF_001507265.1 | Human | Europe | ST-794 | ST-677 complex |
| **143** | GCF_001506925.1 | Human | Europe | ST-794 | ST-677 complex |
| **144** | GCF_001506265.1 | Human | Europe | ST-794 | ST-677 complex |
| **145** | GCF_001506645.1 | Human | Europe | ST-794 | ST-677 complex |
| **146** | GCF_001506585.1 | Human | Europe | ST-794 | ST-677 complex |
| **147** | GCF_001506765.1 | Human | Europe | ST-794 | ST-677 complex |
| **148** | GCF_001506785.1 | Human | Europe | ST-794 | ST-677 complex |
| **149** | GCF_003060765.1 | Poultry | Europe | ST-5843 | - |
| **150** | GCF_002024325.1 | Unknown | Europe | ST-5843 | - |
| **151** | GCF_000934305.1 | Human | Europe | ST-5843 | - |
| **152** | GCF_000493495.1 | Unknown | Europe | ST-45 | ST-45 complex |
| **153** | GCF_003368065.1 | Human | Europe | ST-45 | ST-45 complex |
| **154** | GCF_002209005.1 | Cattle | North America | ST-403 | ST-403 complex |
| **155** | GCF_001457695.1 | Unknown | Europe | ST-403 | ST-403 complex |
| **156** | GCF_001299565.1 | Human | Africa | ST-362 | ST-362 complex |
| **157** | GCF_001299595.1 | Human | Africa | ST-362 | ST-362 complex |
| **158** | GCF_000466065.2 | Human | North America | ST-21 | ST-21 complex |
| **159** | GCF_000466105.2 | Human | North America | ST-21 | ST-21 complex |
| **160** | GCF_000468915.2 | Human | North America | ST-21 | ST-21 complex |
| **161** | GCF_001951295.1 | Environment | Europe | ST-21 | ST-21 complex |
| **162** | GCF_001951315.1 | Environment | Europe | ST-21 | ST-21 complex |
| **163** | GCF_001951335.1 | Environment | Europe | ST-21 | ST-21 complex |
| **164** | GCF_002209025.1 | Human | North America | ST-43 | ST-21 complex |
| **165** | GCF_000009085.1 | Human | Europe | ST-43 | ST-21 complex |
| **166** | GCF_002224325.1 | Human | Europe | ST-43 | ST-21 complex |
| **167** | GCF_002238375.1 | Human | Europe | ST-43 | ST-21 complex |
| **168** | GCF_900475265.1 | Human | Europe | ST-43 | ST-21 complex |
| **169** | GCF_000830865.1 | Unknown | Unknown | ST-43 | ST-21 complex |
| **170** | GCF_000304375.1 | Unknown | Unknown | ST-43 | ST-21 complex |
| **171** | GCF_000830775.1 | Unknown | Unknown | ST-43 | ST-21 complex |
| **172** | GCF_000830805.1 | Unknown | Unknown | ST-43 | ST-21 complex |
| **173** | GCF_000830825.1 | Unknown | Unknown | ST-43 | ST-21 complex |
| **174** | GCF_000830845.1 | Unknown | Unknown | ST-43 | ST-21 complex |

Seventy-six sequences lower than the cut-off value (branch length of 0.00005) were collapsed. Sequences collapsed together were highlighted with different color.
